# Scientific Proceedings of the Richmond Academy of Medicine and Surgery

**Published:** 1890-10

**Authors:** W. W. Parker, J. W. Henson

**Affiliations:** President, in the Chair


### SCIENTIFIC PROCEEDINGS OF THE RICHMOND ACADEMY OF MEDICINE AND SURGERY.

*August 26th, 1890.*

DR. W. W. PARKER, President, in the Chair. J. W. HENSON, M. D., Reporter.

### SYMPTOMS OF COCAINE POISONING, FROM ONE HALF DRACHM (PER RECTUM) OF A 3 PER CENT SOLUTION.

Dr. Ramon D. Garcin reported that six weeks or two months since, he was called to see Mr. F——, who had been operated upon by an irregular, who was out of the city when Dr. G. was called.

When the latter reached the man he was suffering intensely, and morphia hypodermically not relieving him, one half drachm of a 3 per cent solution of cocaine was administered per rectum. No relief of pain ensued but in a short time breathing became quickened—extremities cold—pulse rapid and weak. The man described his flesh as tingling, like the sensation felt upon first grasping the poles of a galvanic battery.

By the use of stimulants he soon rallied.

Dr. J. P. Roy asked if the muscles of deglutition were affected.

Dr. Garcin—No.

### A CASE FOR DIAGNOSIS.

On July 8th, 1890, Dr. Garcin said he was called to see Mr. M., aged 19 years, who complained of intense nausea and pains resembling cramps in the region of the epigastrium. The history of the case before this was negative.

He had been to work up to the day before taking his bed, although a week previously there was a slight diarrhœa for a few days, but when the doctor was called the man said his bowels were in a normal condition.

His tongue was very slightly furred, temperature (by mouth) 98 1-2 F., the abdomen—especially about the umbilical region was very tympanitic—a distinct gurgling, exactly resembling that of typhoid fever, was present in both iliac fossae—the bowels had been moved once that day.

The doctor gave a simple anodyne for the cramp (which soon afforded relief) and dilute hydro-chloric acid—15 drops every 4 hours.

July 9th and 10th.—Patient about the same, bowels acting once daily. The doctor ordered a mixture of equal parts of turpentine and sweet oil over the iliac region. He, from the first, ordered liquid diet. The temperature was normal—taken once that day.

July 12th.—The characteristic diarrhoea of typhoid—"pea soup" discharges—temperature normal morning and evening. Tympanites being more decided—15 drops of turpentine in emulsion every 6 hours was ordered.

July 13th, 14th and 15th.—Tympanites decidedly diminished. Diarrhoea worse towards evening—3, 4 and sometimes 5 discharges from 3 p. m. to 6 or 7 p. m.

The doctor ordered 15 gtts. dilute hydro-chloric acid and 10 grs. each of lactopeptine and bismuth subnitrate, every 4 hours.

July 16th and 17th.—Patient seen by Dr. R. T. Ellis for Dr. G.

July 18th, 19th, 20th and 21st.—Bowels not so bad, tympanites had disappeared, slight gurgling in right iliac fossa—no fever.

July 22.—Pulse and temperature normal, bowels moved once, tongue healthy.

The interesting features of this case, said the doctor, were:

1. Entire absence of fever, morning and evening.
2. Absence of typhoid tongue.
3. Absence of coma—the man being conscious throughout the attack.

The after treatment was a tonic of Vin Mariani. Patient was out by August 1st.

Dr. Parker—Any history of phthisis?

Dr. Garcin—No.

Dr. J. M. Winfree—Any pain, mucus or blood?

Dr. Garcin—Some pain—no mucus or blood.

Dr. T. J. Moore—How long was the man sick?

Dr. Garcin—About 3 weeks.

Dr. Moore—Did Dr. G. see him when first taken?

Dr. Garcin—Yes—when he first took to bed, but had been complaining before—although at work up to the day before the doctor's first visit.

Dr. Moore—What was his work?

Dr. Garcin—Apprenticed lithographer.

Dr. Moore—Did he work in lead?

Dr. Garcin—Yes, a little in mixing colors.

Dr. Moore stated that the symptoms were so obscure, it was impossible to make anything like an accurate diagnosis.

When a person was subjected to the gradual and prolonged absorption of lead, there occurred sometimes a condition where there was no manifestation of colica pictonum proper, but a certain degree of constipation followed by an irritative diarrhoea. *Possibly* this patient was so affected.

The diurnal normal temperature excluded typhoid fever.

We sometimes have a condition of bowel where a local irritation of a diarrhoeic character congests and causes ulceration of Peyer's patches—this might give you the characteristic pultaceous stools with the fetor of typhoid actions accompanied by tympanites.

Mere tympanites occurred in so many conditions that it was not calculated to lead up to a diagnosis.

Tenderness in the ilio-coecal region was more directly prognostic.

Abscess of the parotids complicating typhoid fevers.

Dr. Parker had seen, in a boy aged 16 years, abscess of each parotid gland, as a complication of typhoid. Each discharged from the ear before being lanced—the discharge through the ear ceasing after the lancing.

The point was, that the boy recovered, though some one had stated that all cases of typhoid fever with abscess about the parotid gland proved fatal.

FATALITY IN TYPHOID FEVER WITH ABSCESS OF THE PAROTID GLAND.

Dr. Moore said that several years since, Dr. R. M. C. Page, of New York, wrote an article on secondary parotitis, in which he stated that when suppuration of the parotid gland arose as a complication of typhoid fever, *nearly all* cases so *affected* proved fatal.

Dr. Roy had a similar case to Dr. Parker's, last Fall, occurring about the third week of typhoid. As in Dr. P.'s case, both abscesses discharged from ear. There was also an accompanying cancrum oris—a spot of gangrene the size of a silver dollar appearing on outside of the cheek before *death, which followed soon.*

ANEURISM OF THE ARCH OF THE AORTA.

Dr. Lewis C. Basher was called in consultation with Dr. Jones to see a colored woman who was suffering from the effects of a pulsating tumor, occupying the upper part of the left side of the thorax. He found a patient about 35 years of age who was exceedingly emaciated and suffering greatly from pain, dyspnoea and extreme debility. She had little or no appetite.

On examination, the tumor, which measured about 3x3 1-2 inches at every point gave a distinct pulsation corresponding to the cardiac systole. The stethoscope gave only an indistinct "bruit." The sternum appeared to project forward, and there was a complete dislocation of the left clavicle at the left sterno-clavicular articulation. He (Dr. B.) diagnosed the tumor as an aneurism of the arch of the aorta, which had projected forward, pressing against the sternum, ribs and clavicle, and causing absorption of the former (sternum and ribs) and dislocation of the latter (clavicle.)

On Saturday night last this patient died, and yesterday, with the assistance of Drs. C. A. Blanton, David J. Coleman, Jones and others, a post mortem was made, which confirmed the diagnosis.

A sacculated aneurism, springing from the arch of the aorta and projecting forwards and upwards, had dislocated the left clavicle at its sternal end and had caused absorption of some of the upper ribs, as well as the sternum at the junction of the manubrium and gladiolus. There was a slight rupture in the sac, from which there was probably a slow leakage of blood, thus accounting for the gradual rather than the sudden death such as results from sudden rupture and copious hemorrhage. The left side of the thorax was filled with blood.

The Doctor then exhibited the aneurism to the meeting.

SHOULD SET A LIMIT TO LIFE IN ORGANIC HEART DISEASE WITH CAUTION.

Dr. Parker had reported two or three years ago the case of a man, aged about 75, with enlarged and valvular disease of the heart. Pulse was 24 per minute for months at a time.

While under Dr. P's. observation, a period of two months, he used to apparently die four or five times a day. At the end of said two months he left town, now over two years ago. He just died a few days ago.

A doctor should be careful how he limits life in a person with organic heart trouble. As an illustration the Doctor told of a man named Shook, the action of whose heart (from hypertrophy) was so violent as to shake the bed. After being in bed several months he got up and walked about for one or two years.

MASTOIDITIS IN THE NEGRO.

Dr. W. F. Mercer asked if anybody had ever seen mastoiditis in a full-blooded negro. Dr. T. E. Murrell, of Little Rock, Ark., had stated that mastoiditis was never seen in a full-blooded negro. He (Dr. Mercer) in a dispensary practice of six years, the majority of the patients being negroes too, had never seen a case in a full-blooded negro until within the last two months—one case, a man. His only evidence that he was full-blooded was his appearance and statement.

Dr. Parker had a case in a mulatto. This was operated upon by Dr. J. A. White, but death occurred in three or four weeks afterwards.

CONTINUED FEVERS.

Dr. Parker saw some time ago a case of fever, with Dr. O. A. Crenshaw, who is a great believer in typhoid fever—insisted that this was typhoid for some time, but it was not.

The woman had been badly treated by her husband. Dr. P. thought it an irritative fever. It terminated favorably after three or four weeks duration.

It was not usual, though, to see a continued fever unless it be typhoid. He (Dr. P.) had seen numbers of cases of slow pulse typhoid fever before the war. The amount of prostration, however, proved them to be typhoid, to his mind.

He was now attending a young lady who had had typhoid fever in Charlottesville. Supposed to be decidedly convalescent, she was brought here two weeks ago to escape diphtheria. Moving had done a great deal of harm. Her temperature was now 103 degrees F. She could not walk five steps now without help.

He was called Saturday night to see a remarkably handsome young man in the same house with the young lady. His temperature was 103 degrees, and he presented the symptoms of cold—flushes and steams alternating with cold chills. The Doctor (Dr. P.) thought it a general inflammatory fever, or a sort of general rheumatism. He gave him calomel and soda then, and on Sunday quinine—five grains every four hours.

Monday he was in a profuse cold sweat, pulse feeble, no fever from symptoms (the doctor having left his thermometer behind), bowels and tongue pretty good, appetite bad except for liquids. The Doctor thought he would soon be better.

Tuesday (to-day) his temperature was 103 degrees. Perspiration gone. Skin hot.

Dr. P. forgot to say he had two hemorrhages from the nose Monday night.

He thought the case peculiar, and was uncertain whether it was typhoid or not.

Dr. M. D. Hoge, Jr.—Any cough?

Dr. Parker—None.

Dr. David McCaw—Had Dr. P. given him any antipyrine?

Dr. Parker—None. Had Dr. Moore seen any cases of continued fever not typhoid?

Dr. Moore—Yes.

Dr. Parker—What was the pathology of them?

Dr. Moore stated that every fever was continued in which there was no intermission. For example, remittent malarial fever. What was Dr. Parker's idea of a continued fever?

Dr. Parker thought typhoid and typhus continued fevers, but not malarial fever. Dr. Moore stated that he had seen a form of fever this season that corresponded neither in type nor characteristics to either remittent or typhoid fever. He thought the lines were too sharply drawn by the writers concerning the continued fevers common to various sections of the

United States. We have occasionally in this part of Virginia a form of fever congestive in type (attributable to heat and the peculiar atmospheric conditions), with high and irregular diurnal thermometric ranges, great rapidity of pulse, congestion and tenderness of spleen and liver, congestion of kidneys, a certain amount of tympanites, and of uncertain duration, lasting often from ten days to two weeks. It did not yield to quinine, while alterative doses of mercury modified the disease and shortened the duration of the fever. It was accompanied by a bilious diarrhoea yielding best to bismuth and opium.

Typhoid existed in all parts of the United States. It is most prevalent and violent in type in high altitudes. It hugged the mountains and a belt of Piedmont country contigious thereto. It is also found in the low country and in tertiary formations.

It is modified in many of its symptoms by the effect of prolonged heat and the structural alteration of the glandular organs, particularly the spleen and liver, by malarious influences. The Doctor was not referring to the disease called typho-malarial fever. The cause of typhoid fever has never been ascertained. Scientific men have offered many suggestions without definite results. Sewer gas was often mentioned as the vehicle by which the specific poison is conveyed. In many places, especially mountainous sections, both sewers and sewer gas were unknown. Running and well water were both frequently thought to contain the specific poison.

He did not believe a specific cause or germ had been definitely ascertained. He related how an old Tennessee doctor, unlettered but experienced, at some convention stated that he knew nothing of germs and the other new-fangled notions in regard to the cause of typhoid fever, but that whenever he could induce any of his families to locate their stables and hog pens at a sufficient distance from their houses, and remove their chip piles at the proper seasons, he noticed that such families were not troubled with typhoid fever.

Typhoid differs markedly in regard to the severity of attacks, embracing from the walking cases upon the one hand to the malignant upon the other.

In regard to the perspiration mentioned by Dr. P., he had seen frequent profuse sweats in the first week of inception of typhoid fever, but usually there was a dry skin.

Dr. Parker remarked that in some parts of East Tennessee the people forty years ago had never heard of sewers nor sewer gas, nor of typhoid fever. But he spoke of an old gentleman owning about seventy-five negroes, and who lived seven miles from his nearest neighbor, and the fact that awhile later typhoid broke out among his slaves, although it had not been known of before.

---

*August 12th, 1890.*—Dr. W. W. Parker, President, in the Chair.

The President, Dr. Parker, read a

REPORT OF A POST MORTEM IN A CASE OF OSTEO-SARCOMA.

On the first of January last he saw Mrs. C., aged 24 years (mother of one child of 3 years and now four months pregnant), who had been suffering about two years with a tumor in the right groin. It was nearly the size of the fist, hard and painful on pressure. It appeared (small in size) soon after the birth of her child, and was thought by the doctor then in attendance to be an enlarged gland. Not much attention was paid to it then, as it was not very painful. It grew very gradually, and after two or three months she consulted a surgeon who pronounced it "cancer." This diagnosis shocked her so that she at once took her bed and commenced taking opium. She remained in bed three months in the greatest distress. She finally took heart and got up and attended to her household duties. Several doctors saw her. The tumor was aspirated, and nothing but blood escaped. Electricity was used on the tumor for some time, and seemed to give great relief, but did not reduce its size. She still continued to take opium.

When Dr. Parker first saw her, she was cheerful and quite active, going about the house, and often on the street, though much emaciated. The tumor had been for some time at a about a stand still, and continued so for a good while after he began attendance. The right leg (the right side being the one affected) was not larger than her arm in health—indeed, not so large. The muscles were shrunken and flabby, yet she walked quite well and fast—with a slight limp. The pain was intense down this leg to the heel—sometimes very severe in the calf. She had never been leeched or blistered. The leeching was tried by Dr. P., but no good was done; but small blisters, often repeated, gave great relief. There was a distinct aneurismal thrill in the tumor, which *now* filled the iliac fossa, and was as large as the doctor's fist, but oblong in shape, the smaller end downwards. The thrill proceeded from an artery running obliquely across the tumor outward.

Dr. Parker (it was now about two months since he first saw the woman) called in Dr. Isaiah H. White, to see if this artery might not be tied, thinking it might be feeding the tumor. He thought at least an exploratory incision would be justifiable, provided the lady passed safely through her confinement. She had now been pregnant six months. She was safely delivered at term of a small but healthy infant—chloroform, whisky and ergot being used during the labor. The child still lives and was growing finely.

Six weeks after her confinement she rode out (against Dr. P.'s advice), and came home in great pain, and went to bed. So great was the pain that it was necessary to administer chloroform, as opium would not give relief. She took immense quantities of the former. He forgot to say that about three weeks after her confinement the pain *entirely* disappeared (strange to say). *She* then stopped the opium and chloroform and had strong hopes of complete recovery.

Her appetite, too, became enormous. She ate six hearty meals a day, and would awake at day-break and demand her breakfast. Her appetite reminded him of some convalescents from typhoid fever. It was surprising to see how rapidly her strength and flesh increased. Her spirits rose to the highest pitch. She was the happiest woman the doctor ever saw. No opiate of any sort was now taken.

But in exactly fifteen days the pain returned with great violence, as stated above in connection with the drive after her confinement.

Dr. White again saw her with Dr. Parker, with a view to exploring the tumor. They found it had rapidly increased in size since her confinement—had extended downward, the arterial thrill was now more distinct, and other adjacent vessels were enlarged.

After taking into account the uncertainty of the diagnosis, and that it would be necessary to enter the peritoneal cavity in order to thoroughly explore the tumor, both doctors concluded it would not be wise to attempt an operation. The pain increased to such an extent that sometimes she had to take two drachms of McMunn's elixir of opium every hour.

From the time she rode out until her death (July 27th), she took, in addition to immense quantities of opium, *nineteen pounds* of chloroform. Much of this was wasted, as she often took it herself. Her death was sudden. Upon rising to vomit, she fell upon her pillow and expired without a struggle.

Thirty-six hours after death, in company with Dr. White and Dr. J. Michaux, who had at one time attended her, Dr. Parker laid open the tumor by making two incisions—one over the entire length of the growth downwards and inwards; and a second (small one) across the first, and running inwards. The muscles covering the tumor looked healthy. Upon reaching the mass, he found it impossible on passing his hand inwards, to dislodge it from the brim of the pelvis. It was bound down to the brim as far as the pubes by a dense fibrous tissue—thickened periosteum perhaps. He then withdrew his hand, and passed it outward and backward along the crest of the ilium, coming in contact with an immense amount of diseased bone, which extended backward nearly to the sacrum. In some places he could push his finger nearly through the bone, which was honey-combed. He made an incision into the tumor, and found part of it soft—not exactly medullary, but not at all scirrhous—and of the color of pale muscular tissue.

As the origin of the disease was now plain he did not remove the whole tumor. It evidently began in the bone or the periosteum. The exciting cause may have been the lady's striking her right groin against the edge of a table about four or five years before the trouble began to manifest itself. The accident produced great pain, which lasted for some time.

Dr. Parker said that he read quite a lengthy paper before the Medical Society of Virginia two years ago against the habit of some excellent surgeons who think it their duty to be frank with these patients, and in this paper he took the ground that their course was always unwise in the case of cancer.

This patient was a beautiful woman, full of life and energy, and lost one or two years of comparative happiness by the announcement of the surgeon that the growth was a cancer.

One gratifying result of this post mortem was that it relieved the minds of the husband and mother of the idea that the patient had been neglected or the case mismanaged. It is doubtful if any operation at any time would have been beneficial in its results.

Dr. Michaux had attended this woman in her first confinement, and had her in charge when the tumor first developed. Two and a-half years ago, she came to him complaining of pains in the right iliac region before the appearance of the growth. He examined her per vaginam, and by means of bimanual palpation, suspecting some ovarian trouble, but found no enlargement of that organ or other evidences of disease.

The pains from the beginning came on in paroxysms, which, as time and the case advanced, grew more violent, and the intervals became shorter. Then a small nodule appeared in the right iliac region—growing, but not rapidly. The *length* of the pains increased too. Thinking to learn more, he used the exploring needle. He found a very dense tumor—a few drops of blood (nothing else) escaping. He declined to operate, thinking the growth malignant. Being invited by Dr. Parker, he attended the post mortem, and was still more inclined to think it malignant.

EARLY REMOVAL OF SUSPICIOUS GROWTHS ADVOCATED.

Dr. J. N. Upshur, thought Dr. Parker's case a very interesting one. He had read in some journal a report, in which the writer advocated the removal of suspicious growths before the appearance of any cachexia or involvement of lymphatic glands. He would like to know the result of a microscopical examination, in Dr. Parker's case. The symptoms pointed to malignancy, and the character of the growth, as well as its involvement of bone, suggested the colloid variety.

He didn't agree with Dr. P., in not telling the patient frankly, the prognosis. He thought there was a great deal in the manner of telling.

Break it to the patient so that the shock and horror of it all would be taken off. He had never regretted being frank.

ADENOMA OF THE MAMMARY GLAND.

Dr. Upshur was reminded (by Dr. Michaux's remarks) of the case of a lady whom he had delivered in September last. She had a good labor and a satisfactory "getting up"—except that she had a slight continued fever during the last two weeks of her confinement. She also suffered from sore nipples, for which he had used lead nipple shields. At the seventh week he was called again to see the patient, who was suffering from supposed mammary abscess due, her mother thought, to bruising from the nipple shields. The doctor thought this impossible, as the metal was too soft. After poulticing, he gave chloroform. Fluctuation being as distinct as he had ever felt, he lanced the breast. To his surprise only a little sanguinolent matter was discharged, and the tissue cracked under the knife like a potato. The absence of pus and the peculiar feel on cutting were both very striking features. The pain and throbbing subsided, and the part not discharging, the poulticing was stopped after awhile. The pain and throbbing recommencing, the doctor went through the same process of poulticing and lancing with the same experience, except there was this time some pus. Later still he repeated the above treatment, and this time, upon continuing the poulticing, the place discharged more pus than it had done at all. He stated to the patient's mother that this was not ordinary mammary abscess, but an adenoma, being benign at the time, but that it might in the future degenerate so as to need surgical interference.

After this, under the influence of tonics, the patient's general health improved. He used potassium iodide and fluid extract gentian internally, and five per cent. oleate of mercury locally; but not succeeding in reducing the tumor, after fair trial, the treatment was discontinued, and the patient urgently advised not to rub or handle the gland. She was now put on the syrup of lactophosphate of lime with very decided improvement in her general health. The baby had not been weaned from this breast because of the free flow of milk from the inferior part of the gland, and the fear of abscess in that location; but so soon as the milk could be dried up, the weaning from this breast was done. When the patient passed from under Dr. Upshur's care, she was looking as well as he had ever seen her

She had subsequently an attack of *la grippe*, and a few weeks later, cholera morbus.

The said patient is now in Dr. Hugh M. Taylor's hands, and he (Dr. Upshur) thought the subsequent history of the case would be of interest to the Academy. He had erred, said the doctor, in two points.

First. He believed that if he had stated frankly the probable outcome of the trouble and had advised excision, the woman's life would have been either saved or prolonged.

Secondly. In the light of subsequent history, he believed he erred in lancing the breast, as no good had resulted.

This was a case which illustrated the importance of frankness and early excision. He thought the latter very important in the case of suspicious growths.

SCIRRHUS OF MAMMARY GLAND.

Dr. Upshur also referred to an old lady of sixty. She had in one mammary gland an irregular hard lump, but her former physician (now dead) advised her to let it alone, because, at that time, it gave her no trouble.

Some time ago she sent for Dr. Upshur, and showed him the lump. She complained of trouble in using her right arm, and tenderness of the breast. There were darting pains and *retracted nipple.* The lump was hard and irregular, though not adherent to the chest wall, and presented symptoms of scirrhus. He advised removal. He could not convince her and her friends of the nature of the trouble. He told her frankly if she waited too long it would be too late. [Caution urged in lancing the mammary gland.]

Dr. Parker thought Dr. Upshur wrong in lancing in the first case he reported. This should never be done unless there was absolutely distinct fluctuation. He was suspicious of breast tumors any way unless he could be absolutely sure of abscess. He thought Dr. Upshur also wrong in telling the patient the diagnosis in the case of malignancy. He had as lief shoot a woman as tell her she was cancerous. Tell her friends always but not the patient. Thousands and thousands were made miserable by this practice. He was sustained, said the doctor, by Mr. Locke.

Dr. Upshur stated that the fluctuation was positive and unmistakable. He believed he would be in error to state that fluctuation and the peculiar feeling in cutting, before described, when taken together, were characteristic of malignancy.

Dr. Hugh M. Taylor said the history of the first case reported by Dr. Upshur, had been given him just about as the doctor had related it. He refused to say anything about the case, as the patient and friends being very sensitive, preferred that it should not be spoken of.

THE DIAGNOSIS OF SCIRRHUS OF THE RECTUM IN A CHILD OF 13 YEARS CONFIRMED.

Dr. Michaux referred to the case of scirrhus of the rectum in a child of 13 years, reported by him at the meeting of this body July 8th. Since that report he called Dr. J. S. D. Cullen in consultation, who confirmed his (Dr. Michaux's) diagnosis. The growth finally closed up the calibre of the bowel, but his family would not submit to artificial relief. The child died, the doctor thought, more from lack of drainage through the bowels than from sheer malignancy. He had only one action in fourteen days. That was just before his death. It was small in quantity, consisting of a little mucus and blood, with a very small amount of fluid fæcal matter. He thought there could now be no doubt as to the malignancy of the trouble.

POINT OF SELECTION FOR MAKING ARTIFICIAL ANUS.

Dr. M. D. Hoge, Jr., had read Dr. Hunter McGuire's report on twenty-one cases of supra-pubic cystotomy, in which Dr. McGuire said that if he had a case upon which to operate for making an artificial anus, he would select somewhere in one of the recti muscles, near the median line, because he had noticed that the construction of these muscles tended to close any opening that might be about the location just mentioned. This case of Dr. Michaux's would have been a good one for such an operation. The boy's life might have been prolonged if such a step could have been taken after the bowel was closed.

### OSTEO-SARCOMA OF THE ORBIT.

Dr. Chas. M. Shields, three months ago, saw a child eighteen months old suffering from protopsis of the right eye-ball. It began as a drooping of the upper lid. Ptosis soon gave place to protrusion of the ball. At the time he first saw it (five weeks after its commencement), the right eye-ball was about three-quarters of an inch in advance of its fellow. The cause seemed to be some growth or enlargement situated about the upper and outer part of the orbit. The said growth or enlargement continued to increase in an upward and outward direction for a period of three weeks, when the doctor advised an operation. The original trouble seeming as above stated, to be at the upper and outer part of the eye, made him suspect a cancerous enlargement of the lachrymal gland.

He went with an assistant to the patient's home, intending to put it under the influence of chloroform and examine the part, also to draw some fluid from the suspected gland for microscopic examination. He found the child, however, had a fever, and he turned the patient over to the family physician.

In a few days the said physician called in Dr. Shields, who then found the child had decided meningitis, with high fever and marked opisthotonos. There was also purpura hæmorrhagica over the whole surface of the body. The child died.

The day before death there was a hæmorrhage from the nose, which the doctor attributed to extension of the growth into the ethmoid bone.

A post-mortem was held. A tumor had begun from the roof at the upper and outer part of the orbit. This fact, and its growing upward and outward, had originated the idea of cancer of the lachrymal gland. The growth extended over into the ethmoid, and involved it considerably. He had sent a specimen to the army and navy bureau for microscopical examination. He had gotten no report. He thought the growth an osteo-sarcoma. The most peculiar feature about the whole affair was the purpura hæmorrhagica of the whole surface of the body.

